# Association of systemic inflammatory indices and body mass index with diabetic macular edema in non-proliferative diabetic retinopathy

**DOI:** 10.1186/s12886-026-04906-6

**Published:** 2026-05-27

**Authors:** Ohisa Harley, Yufilia Suci Amelia, Elsa Gustianty, Nanny N. M. Soetedjo, Arief S. Kartasasmita

**Affiliations:** 1 Doctoral Program in Medical Sciences, Faculty of Medicine, Padjadjaran University, Bandung, West Java Indonesia; 2 Netra Eye Clinic Centre, Bandung, West Java Indonesia; 3 Unpad Hospital, Jatinangor, West Java Indonesia; 4 Department of Ophthalmology, Faculty of Medicine, Padjadjaran University, Bandung, West Java Indonesia; 5 Cicendo National Eye Hospital Center, Bandung, West Java Indonesia; 6 Department of Endocrinology and Internal Medicine, Faculty of Medicine, Padjadjaran University, Bandung, West Java Indonesia

**Keywords:** Diabetic macular edema, Systemic inflammation, NLR, SII, BMI, Diabetic retinopathy, Biomarkers

## Abstract

**Purpose:**

To evaluate the association between systemic biomarkers and the presence of diabetic macular edema (DME) with non-proliferative diabetic retinopathy (NPDR), and to assess the discriminative ability of these markers for identifying DME.

**Methods:**

A cross-sectional study was conducted among 80 patients with type 2 diabetes mellitus and NPDR. Participants were categorized into DME and non-DME groups based on spectral-domain optical coherence tomography (OCT), with a central macular thickness ≥ 300 μm defining DME. Hematological parameters were analyzed from complete blood counts. Statistical analysis was performed using SPSS 23.0.

**Results:**

A total of 80 eyes (40 with DME and 40 without DME) were analyzed. Significant elevation of neutrophil counts was observed in the DME group (*p* = 0.006). Similarly, NLR and SII values were higher in the DME group (*p* = 0.075 and *p* = 0.048, respectively). The combined model of NLR, SII, and BMI showed moderate discriminatory ability (AUC 0.706, *p* = 0.002). NPDR severity was significantly associated with DME in the univariate and multivariate analyses of the initial model (OR 3.023, *p* = 0.049). This association lost statistical significance after adjustment for age and BMI. Systemic inflammatory markers (NLR, MLR, PLR, and SII) were not significantly associated with DME.

**Conclusions:**

SII and NLR demonstrated moderate discriminatory ability for identifying DME in patients with NPDR, particularly when integrated with the metabolic parameter BMI. These findings provide foundational evidence for the potential of using an accessible, cost-effective marker in diabetic retinal disease.

## Introduction

Diabetic macular edema (DME) is a major cause of visual impairment among individuals with diabetic retinopathy (DR) [1]. Despite the widespread use of anti–vascular endothelial growth factor (anti-VEGF) therapy, delayed detection and the lack of cost-effective screening strategies continue to limit early management—particularly in low- and middle-income regions where access to advanced imaging modalities remains limited [2, 3]. Identifying simple and affordable systemic biomarkers that can predict DME risk before structural retinal damage occurs is therefore a critical unmet need in diabetic eye care.

The development of DME is driven by chronic hyperglycemia-induced oxidative stress, endothelial dysfunction, and persistent low-grade inflammation [4]. These systemic processes promote the breakdown of the blood–retinal barrier, resulting in vascular leakage and macular thickening. Accumulating evidence suggests that DR and DME are not purely local retinal conditions but systemic inflammatory microangiopathies reflecting generalized vascular injury [4–7]. This understanding provides the rationale for exploring hematologic inflammatory indices as potential markers of retinal complications.

Our previous meta-analysis showed significant associations between the elevated neutrophil-to-lymphocyte ratio (NLR), platelet-to-lymphocyte ratio (PLR), and systemic immune-inflammation index (SII) and the presence and NPDR Severity, especially in proliferative stages [8]. However, it remains uncertain whether these basic inflammatory markers can independently differentiate diabetic macular edema (DME) from proliferative disease. As DME frequently occurs during the non-proliferative phase, it is essential to identify the early stage to facilitate timely management.

This study aimed to evaluate the relationship between systemic inflammatory indices (NLR, PLR, SII, and monocyte-to-lymphocyte ratio [MLR]), and diabetic macular edema (DME) in patients with non-proliferative diabetic retinopathy (NPDR), and to assess the discriminative ability of these markers for identifying DME. By integrating routinely available CBC-derived data, the study seeks to establish an effective, low-cost examination for DME that can be applied in resource-limited clinical settings, ultimately enabling earlier detection and prevention of vision-threatening disease.

## Methods

### Study design and ethical approval

This cross-sectional comparative study was conducted between March and August 2025. The protocol adhered to the tenets of the *Declaration of Helsinki* and was approved by the Ethics Committee of Padjadjaran University (No. 85/UN6.KEP/EC/2025). Written informed consent was obtained from all participants before enrollment.

### Study population

Eligible subjects were adults (≥ 18 years) with type 2 diabetes mellitus (T2DM) diagnosed by an internist and confirmed *non-proliferative diabetic retinopathy (NPDR)* on fundus examination. Exclusion criteria included active systemic infection or inflammatory disease, autoimmune disorders, malignancy, renal failure, recent surgery, or ocular treatment within the preceding 1 month, such as panretinal photocoagulation (PRP), intravitreal injection, or vitrectomy.

Patients with proliferative diabetic retinopathy (PDR) were intentionally excluded to maintain a more homogeneous disease stage and to minimize potential confounding from advanced ischemia and neovascular processes characteristic of PDR, which may independently influence systemic inflammatory markers.

### Examination and laboratory assessment

Comprehensive ophthalmic assessment included best-corrected visual acuity, slit-lamp biomicroscopy, and fundus photography (CLARUS 500, Zeiss). Spectral-domain optical coherence tomography (OCT, CIRRUS HD-OCT, Zeiss) was performed to measure central macular thickness (CMT) as the distance between the internal limiting membrane and retinal pigment epithelium. The mean value of the OCT measurements was used for statistical analysis. Diabetic macular edema (DME) was defined as a CMT ≥ 300 μm in at least one eye. In cases where DME was present bilaterally, the eye with the higher CMT value (“worse eye”) was included for analysis to avoid inter-eye correlation bias.

NPDR severity was graded according to the Early Treatment Diabetic Retinopathy Study (ETDRS) classification based on fundus examination findings [9]. The grading and OCT evaluations were independently performed by two masked vitreoretinal specialists who were blinded to the patients’ systemic clinical data.

Venous blood samples (5 mL) were collected for routine laboratory testing, including complete blood count (CBC), free blood glucose (mg/dl), and glycated hemoglobin (HbA1c) (%). Inflammatory indices were derived from CBC parameters as follows:

- NLR = neutrophil count / lymphocyte count
- PLR = platelet count / lymphocyte count
- MLR = monocyte count / lymphocyte count
- SII = (neutrophil × platelet) / lymphocyte count

Body mass index (BMI) was calculated as weight (kg) divided by height squared (m²). These hematologic ratios were selected for their potential to reflect systemic inflammatory and immune responses associated with diabetic microvascular complications.

### Statistical analysis

The normality of continuous variables was assessed using the Shapiro–Wilk test. Continuous variables were expressed as mean ± standard deviation and compared using the independent *t*-test or Mann–Whitney *U*-test as appropriate. Categorical variables were analyzed using the Chi-square or Fisher’s exact test. Logistic regression analyses were performed to determine associations between systemic markers and DME, expressed as odds ratios (OR) with 95% confidence intervals (CI).

Receiver operating characteristic (ROC) analysis was performed to evaluate the discriminatory ability of NLR, SII, and BMI in differentiating between study groups. Optimal cut-off values were determined using the Youden index and were considered exploratory thresholds within this dataset. The discriminatory performance of each marker was evaluated using the area under the ROC curve (AUC). The AUC values were interpreted according to commonly used thresholds as follows: [10]

- AUC = 0.5: no discriminatory ability (equivalent to random classification)
- 0.5 < AUC ≤ 0.6: poor discrimination
- 0.6 < AUC ≤ 0.7: moderate discrimination
- 0.7 < AUC ≤ 0.8: good discrimination
- 0.8 < AUC ≤ 0.9: excellent discrimination
- AUC > 0.9: outstanding discrimination

A combined ROC analysis model was subsequently tested, and model discrimination was compared. A *p*-value < 0.05 was considered statistically significant. Based on these cut-off values, variables were dichotomized and subsequently entered into logistic regression models.

To evaluate the association between systemic inflammatory and metabolic parameters and the presence of diabetic macular edema (DME), univariate logistic regression analysis was first performed. The variables analyzed included inflammatory markers (NLR, PLR, MLR, and SII), the metabolic parameter body mass index (BMI), and potential clinical factors that might influence the outcome, including glycated hemoglobin (HbA1c) and the severity of diabetic retinopathy. Variables with a p-value < 0.20 in the univariate analysis were subsequently included in the multivariate logistic regression model. Prior to multivariable modeling, multicollinearity among variables was assessed using the variance inflation factor (VIF), and variables with VIF < 5 were considered acceptable for inclusion in the multivariate model. Statistical significance in the multivariate analysis was defined as *p* < 0.05. Results were presented as odds ratios (OR) with 95% confidence intervals (CI).

Duration of diabetes was not included in the regression analysis because it was based on patient-reported history, which may be subject to recall bias and could introduce subjectivity in this cross-sectional study.

## Results

### Baseline characteristics

A total of 80 eyes from 80 patients with non-proliferative diabetic retinopathy (NPDR) were included, comprising 40 eyes with diabetic macular edema (DME) and 40 without. The mean age of total participants was 61.1 ± 8.3 years, and the mean duration of diabetes was 11.3 ± 7.8 years. No significant differences were observed between groups in age, diabetes duration, and intraocular pressure (*p* > 0.05). The mean best-corrected visual acuity was significantly worse in the DME group (0.53 ± 0.40 logMAR) compared with the non-DME group (0.17 ± 0.17 logMAR; *p* < 0.001). The NPDR Severity varied significantly between groups, with a higher proportion of severe NPDR in the DME group than in the non-DME group (*p* = 0.039).

Regarding prior ocular treatment, a small number of patients in the DME group had received intravitreal anti-VEGF therapy, while some patients in both groups had a history of PRP laser treatment.

**Fig. 1:**
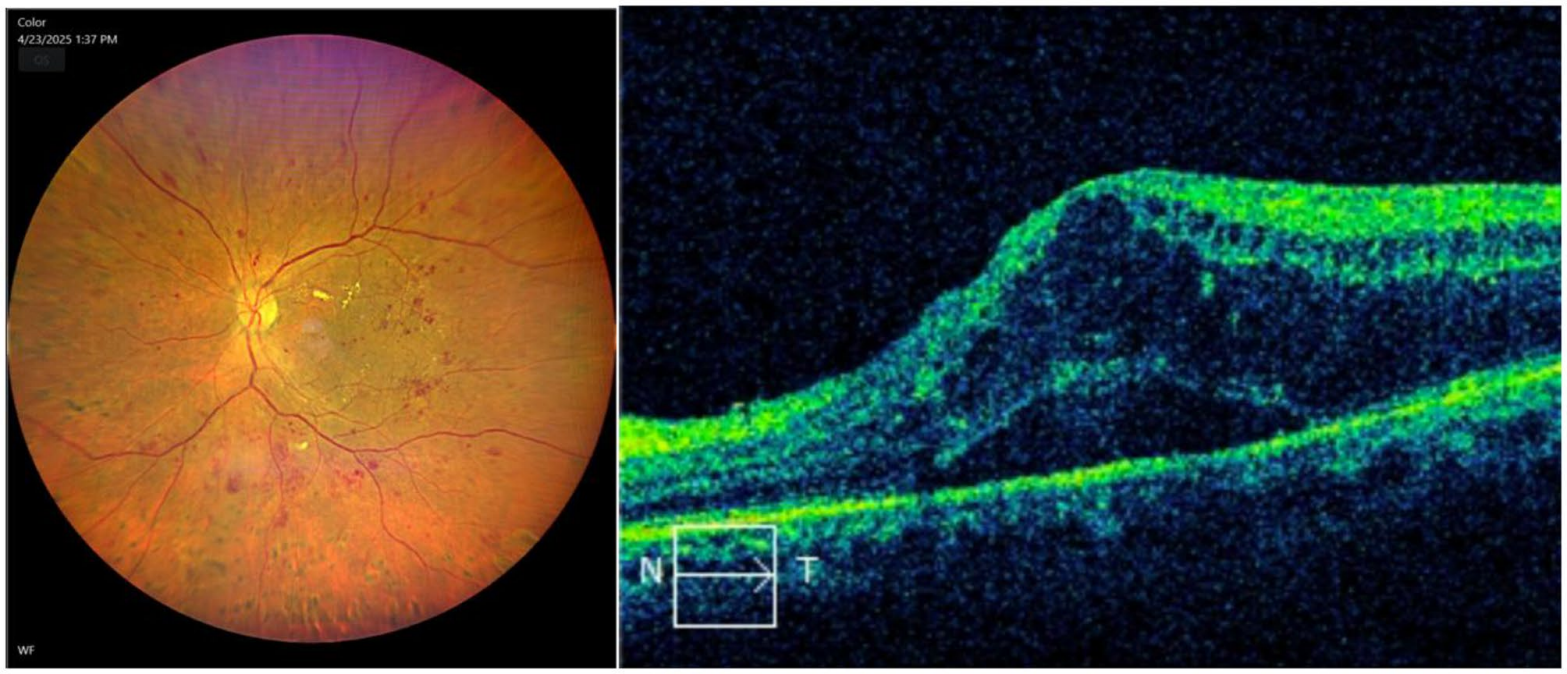
Fundus photograph and OCT examination in DME patients

The mean CMT was 396.3 ± 124.8 μm in the DME group and 248.5 ± 26.7 μm in the non-DME group (*p* < 0.001). Detailed baseline characteristics are provided in Table 1, and the characteristics of DME and NPDR are presented in Fig. 1.

**Table 1:** Baseline characteristics

Variable			DME *N* = 40	Non DME *N* = 40	*P* value
Age (years)			59.5 ± 8.20	62.8 ± 8.20	0.076
Visual acuity (logMAR)			0.53 ± 0.40	0.17 ± 0.17	< 0.001**
Intraocular pressure (mmHg)			15.25 ± 3.01	16.23 ± 2.92	0.146
Duration of diabetes (year)			9.35 ± 5.63	13.15 ± 9.23	0.074
Sex					
Male			17	12	0.244
Female			23	28	
NPDR Severity					
Mild-Moderate			6	14	0.039*
Severe			34	26	
History of treatment^+^					
anti VEGF intravitreal			2	0	
PRP Laser			8	11	
History of systemic disease^+^					
Hypertension			21	22	
Nephropathy			2	1	
Neuropathy			0	4	
Cardiovascular disease			3	6	
Central macular thickness (µm)			396.28 ± 124.75	248.45 ± 26.66	< 0.001**

Categorical data were analyzed using the chi-square test, and continuous data were analyzed using the independent samples t-test. *p-value < 0,05; **p < 0.001

^+^ Data regarding ocular treatment history (conducted > 1 month prior to enrollment) and systemic comorbidities are presented descriptively. The numbers shown in the table may include multiple comorbidities or a history of multiple treatments. None of the patients in this study had a history of vitrectomy. These variables were not included in comparative statistical analyses

DME = diabetic macular edema; NPDR = non-proliferative diabetic retinopathy; Anti-VEGF = anti-vascular endothelial growth factor; PRP = panretinal photocoagulation; BCVA = best-corrected visual acuity

### Metabolic and hematologic inflammatory indices

Baseline metabolic and hematologic parameters are presented in Table 2. Body mass index (BMI), free blood glucose (FBG), and HbA1c levels did not differ significantly between the DME and non-DME groups.

Complete blood count parameters were subsequently evaluated. Platelet counts were comparable between the two groups. However, neutrophil counts were significantly higher in the DME group compared with the non-DME group (*p* = 0.006). Total leukocyte counts were also significantly elevated in the DME group (*p* = 0.016). In contrast, lymphocyte and monocyte counts did not differ significantly between groups (Table 2).

Among the derived inflammatory markers, SII was significantly higher in the DME group (712,926 ± 451,533 vs. 574,671 ± 268,821; *p* = 0.048). Although NLR, PLR, and MLR were numerically elevated in DME, their differences did not reach statistical significance (all *p* > 0.05) (Table 3).

**Table 2:** Clinical and laboratory markers of DME and Non-DME group

Variable		Total *N* = 80	DME *N* = 40	Non DME *N* = 40	*P* value
BMI (kg/m^2^)		24.57 ± 3.5	24.64 ± 3.00	24.71 ± 3.83	0.94
Free blood glucose (mg/dl)		195.24 ± 61.08	184.53 ± 63.02	205.95 ± 57.89	0.05
HbA1C (%)		8.42 ± 2.12	8.05 ± 2.19	8.80 ± 2.01	0.055
Complete blood count					
Platelet (×10^3^/µL)		314.56 ± 96.72	309.69 ± 84.42	298.05 ± 66.09	0.95
Neutrophil absolute (×10^3^/µL)		8.51 ± 2.69	8.94 ± 2.27	8.08 ± 3.02	0.006*
Leukocyte absolute (×10^3^/µL)		5.38 ± 2.12	5.74 ± 1.72	5.02 ± 2.42	0.016*
Lymphocytes absolute (×10^3^/µL)		2.81 ± 0.82	2.84 ± 0.84	2.78 ± 0.81	0.73
Monocyte absolute (×10^3^/µL)		0.33 ± 0.19	0.36 ± 0.22	0.29 ± 0.15	0.19

Categorical data were analyzed using the chi-square test, and continuous data were analyzed using the independent samples t-test. *p-value < 0,05

BMI = body mass index; HbA1c = glycated hemoglobin

**Table 3:** Inflammatory marker profile of DME and Non-DME group

Inflammatory Marker	Total *N* = 80	DME *N* = 40	Non DME *N* = 40	*P* value
NLR	2.02 ± 0.79	2.16 ± 0.86	1.87 ± 0.68	0.075
MLR	0.12 ± 0.07	0.13 ± 0.08	0.10 ± 0.05	0.829
PLR	120.18 ± 48.83	124 ± 58.97	116.37 ± 35.15	0.154
SII	643,798.80 ± 375,719.38	712,926.27 ± 451,533.51	574,671.32 ± 268,821.37	0.048*

Continuous data were analyzed using the independent samples t-test. *p-value < 0,05

DME = Diabetic macular edema; NLR = neutrophil-to-lymphocyte ratio; PLR = platelet-to-lymphocyte ratio; MLR = monocyte-to-lymphocyte ratio; SII = systemic immune-inflammation index

### Diagnostic performance of systemiciinflammatory markers for detecting DME

ROC analysis demonstrated that both NLR and SII had moderate discriminatory ability for identifying DME, with AUC values of 0.638 (95% CI 0.515–0.760, *p* = 0.034) and 0.650 (95% CI 0.529–0.771, *p* = 0.021), respectively. The optimal cut-off points, determined by Youden’s index, were NLR > 2.25 and SII > 558,596, corresponding to sensitivities of 42.5% and 65%, respectively, and specificities of 85% and 65%. The PLR and MLR showed low sensitivity levels (20% and 15%, respectively) and were not statistically significant in differentiating DME. (*p* > 0.05). Additionally, a BMI and HbA1C had poor discriminative ability (Table 4).

**Fig. 2:**
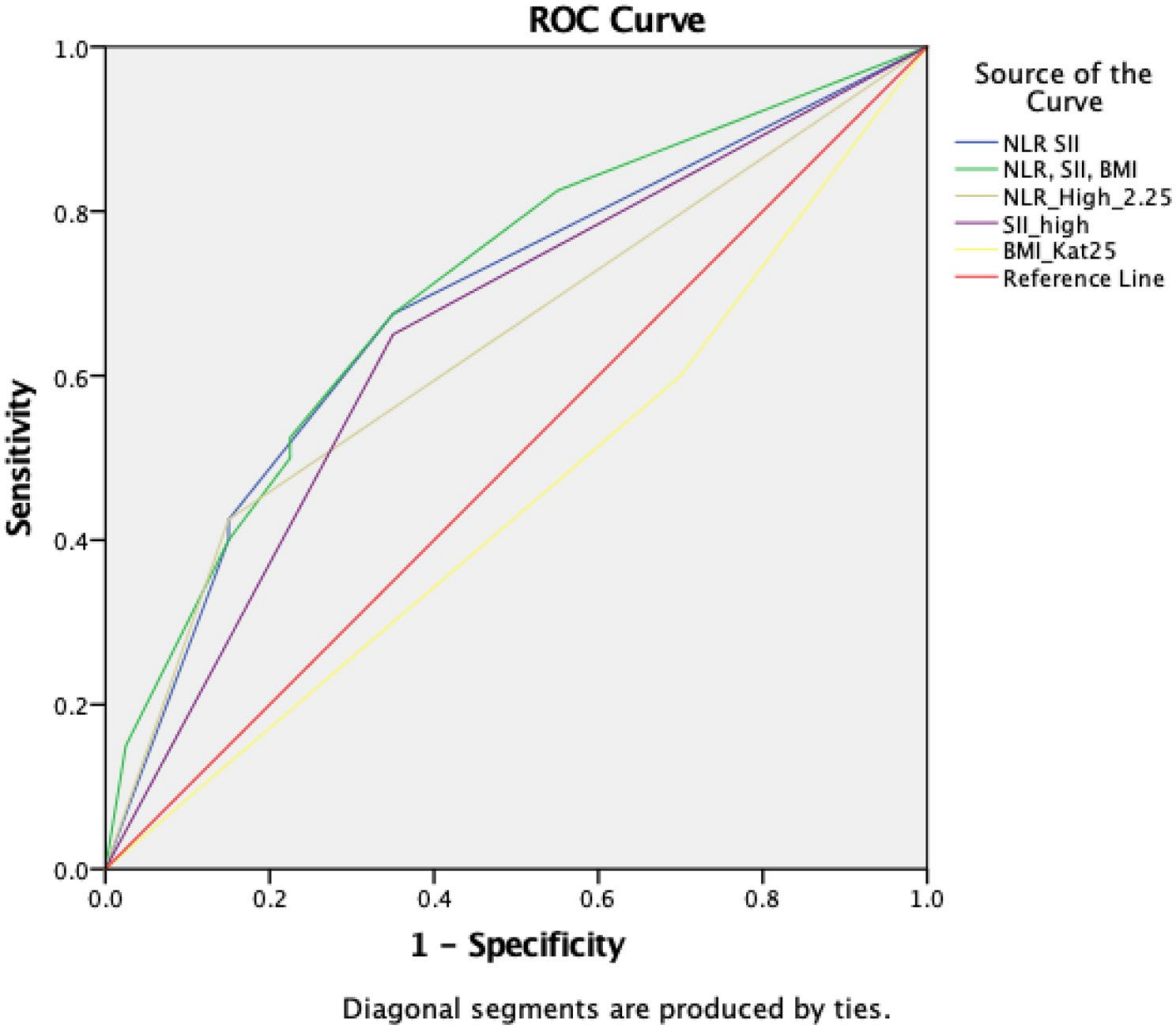
ROC curves showing the performance of individual and combined biomarker models in DME

A combined biomarker model was constructed using the ROC-derived cut-off values. (Table 5; Fig. 2) The combination of NLR ≥ 2.25 and SII ≥ 558,596 yielded an AUC of 0.684 (*p* = 0.005). When BMI ≥ 25 kg/m² was added to the model, the AUC increased to 0.706 (*p* = 0.002).

**Table 4:** Diagnostic performance of systemic inflammatory markers for detecting DME based on ROC analysis

Biomarker	Cut off Value	Sensitivity	Specificity	AUC	95% CI		*P* value
					Lower	Upper	
NLR	2.25	42.5%	85%	0.638	0.515	0.760	0.034*
PLR	154	20%	92.5%	0.563	0.436	0.689	0.336
MLR	0.204	15%	97.5%	0.563	0.436	0.689	0.336
SII	558,596	65%	65%	0.650	0.529	0.771	0.021*
BMI (kg/m^2^)	25	40%	68%	0.450	0.323	0.577	0.441
HbA1C (%)	12.5	7.5%	95%	0.513	0.385	0.640	0.847

*p-value < 0,05

AUC = area under the curve; 95% CI = 95% confidence interval; DM= diabetes melitus; DME = Diabetic macular edema; MLR = monocyte-to-lymphocyte ratio; NLR = neutrophil-to-lymphocyte ratio; PLR = platelet-to-lymphocyte ratio; ROC = receiver operating characteristic; SII = systemic immune-inflammation index

**Table 5:** Diagnostic model integrating systemic biomarkers for DME based on ROC analysis

Biomarker	AUC	*P* value
NLR ≥ 2.25 + SII ≥ 558,596	0.684	0.005
NLR ≥ 2.25 + SII ≥ 558,596 + BMI ≥ 25	0.706	0.002

*p-value < 0,05

AUC = Area Under the Curve; 95% CI = 95% confidence interval; BMI= Body Mass Index; NLR = neutrophil-to-lymphocyte ratio; SII = systemic immune-inflammation index

### Logistic regression approach

A univariate logistic regression analysis was performed to identify factors associated with the presence of DME in patients with NPDR. Based on the results (in Table 6), only NPDR Severity was significantly associated with DME, with an OR of 3.051 (95% CI = 1.032–9.022, *p* = 0.044). Other variables, including inflammatory markers (NLR, PLR, MLR, and SII) and clinical parameters (HbA1c and BMI), did not show a statistically significant association in the univariate model (*p* > 0.05).

Variables with clinical relevance were subsequently entered into multivariate logistic regression models (Table 7). In Model 1, which included NPDR severity, NLR, SII, and HbA1c, NPDR Severity remained significantly associated with DME (OR 3.023, 95% CI = 1.007–9.080, *p* = 0.049). However, in Model 2, after adjusting for age and BMI, none of the variables remained statistically significant.

These findings indicate that while NPDR severity is a notable risk factor for DME, its independent association was not confirmed after full multivariable adjustment in the adjusted multivariate model.

**Table 6:** Univariate logistic regression analysis of factors associated with DME

Variable	Univariate Logistic Regression		
	OR	95% CI	*P* value
NLR	1.685	0.905–3.139	0.100
PLR	1.003	0.994–1.013	0.482
MLR	565.807	0.453–707,323.92	0.081
SII	1.00	1.001–1. 002	0.122
HbA1C	0.841	0.676–1.046	0.120
BMI	0.994	0.832–1.188	0.948
NPDR Severity	3.051	1.032–9.022	0.044*

*p-value < 0,05

95% CI = 95% confidence interval; BMI= Body Mass Index; DM= diabetes melitus; MLR = monocyte-to-lymphocyte ratio; NLR = neutrophil-to-lymphocyte ratio; PLR = platelet-to-lymphocyte ratio; ROC = receiver operating characteristic; SII = systemic immune-inflammation index

**Table 7:** Multivariate logistic regression analysis of factors associated with DME

Variable	Model 1			Model 2		
	*P*	OR	95% CI	*P*	OR	95% CI
Age	-	-	-	0.112	0.940	0.871–1.015
BMI	-	-	-	0.623	0.955	0.793–1.149
NPDR Severity	0.049*	3.023	1.007–9.080	0.968	1.038	0.165–6.546
NLR	0.824	1.121	0.412–3.050	0.898	1.110	0.228–5.401
SII	0.135	1.00	1.00-1.001	0.501	1.00	1.00–1.001
HbA1C	0.156	0.842	0.665–1.068	0.318	0.862	0.643–1.154

*p < 0.05; Continuous variables (age, BMI, NLR, SII, and HbA1c) were analyzed as continuous variables

Model 1: multivariable logistic regression including NPDR Severity, NLR, SII, and HbA1c

Model 2: multivariable logistic regression adjusted for age and BMI

NLR = neutrophil-to-lymphocyte ratio; NPDR: non-proliferative diabetic retinopathy; SII = systemic immune-inflammation index

## Discussion

This study evaluated the relationship between systemic inflammatory indices, particularly the NLR and the SII, and the metabolic parameter BMI in relation to the presence of DME among patients with NPDR. Although this study did not demonstrate a clear independent association between systemic inflammatory and metabolic markers and the occurrence of DME after multivariable adjustment, the findings indicate that NLR and SII showed moderate discriminatory ability in identifying DME in patients with NPDR. Patients with DME tended to exhibit higher systemic inflammatory profiles, particularly reflected by elevated SII, neutrophil, and leukocyte counts. Our study also suggests that systemic inflammatory activity, particularly at certain cut-off values, may reflect the systemic inflammatory activity associated with macular edema in diabetic retinal disease.

Our previous meta-analysis demonstrated that systemic inflammatory indices such as NLR, SII, and PLR are associated with diabetic retinopathy, with higher levels observed in more severe stages of the disease [11]. This underscores the importance of differentiating NPDR from proliferative stages when evaluating systemic inflammatory markers in diabetic retinal disease [12]. The present study focused specifically on NPDR to better elucidate inflammatory alterations associated with DME at an earlier stage of diabetic retinal disease. In addition, Bhutia et al. and Figueras et al. mainly evaluated circulating cytokines such as interleukins, whereas the present study assessed inflammatory indices derived from routine complete blood count parameters, which may provide more accessible indicators of systemic inflammation in clinical practice [13, 14]. Additionally, we demonstrated that the combination of systemic inflammatory and metabolic markers may improve the ability to distinguish DME in patients with NPDR.

SII may reflect inflammatory activity in DME because it integrates neutrophil, lymphocyte, and platelet counts, thereby capturing interactions between the inflammatory response and thrombo-inflammatory activation [15, 16]. Increased neutrophil counts indicate activation of innate immune pathways, while relative lymphopenia reflects impaired adaptive immune regulation [17, 18]. Platelets contribute to endothelial dysfunction through the release of inflammatory mediators, including VEGF and platelet-derived growth factor [18–20]. These mechanisms may promote vascular permeability and contribute to disruption of the blood–retinal barrier, a hallmark of DME [21, 22]. The previous study conducted by Wang et al. demonstrated an association between DR and SII; [23] however, in our study, DME in NPDR patients supports a discriminatory ability but does not show an association].

NLR is widely recognized as a simple indicator of the balance between innate and adaptive immune responses. Increased NLR has been associated with endothelial dysfunction and microvascular injury in several chronic metabolic diseases. In the present study supported by Ilhan C et al., [24] NLR showed a tendency toward higher values in patients with DME, suggesting that systemic inflammatory activation may contribute to the inflammatory environment associated with retinal microvascular damage and may reflect early microvascular changes related to DME.

When systemic inflammatory markers were evaluated alongside the metabolic parameter BMI, a moderate improvement in discriminative performance was observed [16]. Adipose tissue is increasingly recognized as an active endocrine organ that produces inflammatory cytokines and adipokines contributing to insulin resistance, chronic low-grade inflammation, and vascular dysfunction [19, 25–27]. Although BMI alone showed limited ability to distinguish DME, the combined NLR–SII–BMI model demonstrated improved performance. This indicates that BMI may reflect the broader metabolic state that interacts with systemic inflammation. Combining metabolic and inflammatory markers could therefore provide a more comprehensive view of the systemic conditions linked to diabetic microvascular complications [16]. However, the magnitude of improvement remained limited, indicating that these markers should be interpreted as complementary indicators rather than definitive diagnostic tools.

In contrast, other systemic blood markers evaluated in this study, including the MLR, PLR, and HbA1c, did not demonstrate a significant association with DME and were insufficient to differentiate between patients with and without DME. Differences in their biological roles may explain these findings. MLR is considered to reflect chronic macrophage-driven inflammation rather than acute endothelial activation. Because monocyte differentiation into macrophages or microglia primarily occurs within retinal tissue, peripheral MLR may not sufficiently reflect the initial inflammatory processes in DME, as also evidenced by other studies studies [28, 29]. Similarly, PLR reflects platelet–lymphocyte interaction but relies on absolute platelet counts rather than platelet activation, making it susceptible to confounding by medications, metabolic status, and other systemic factors [29, 30]. Furthermore, our prior research using OCT has indicated that systemic inflammatory markers, such as PLR, are associated with the presence of hard exudates as OCT features of DME [31–33]. These observations highlight the possible correlation between systemic inflammatory status and structural alterations of the retina. In addition, the exclusion of proliferative DR in this study may have reduced systemic inflammatory variability, potentially limiting the sensitivity of MLR and PLR in this study.

The initial association between NPDR severity and DME in the unadjusted model suggests that more advanced retinal disease may be linked to increased vascular permeability. This attenuation suggests NPDR severity’s effect may be influenced by confounders like age and metabolic status. NPDR severity alone may not be an independent DME predictor but part of a broader systemic and ocular interplay. From a clinical perspective, NLR, SII, and BMI are derived from routine laboratory and anthropometric measurements and therefore may provide accessible indicators of systemic inflammatory and metabolic status in patients with NPDR. In this study, the combined evaluation of these markers demonstrated moderate discriminative ability in distinguishing patients with DME from those without. This may be particularly useful in resource-limited settings where advanced retinal imaging is not readily available. These biomarkers, therefore, may complement, rather than replace, established DME examinations.

The findings of this study should be interpreted in light of several limitations. First, the cross-sectional design limits the ability to determine temporal or causal relationships between systemic inflammation and the development of DME. Second, the relatively modest sample size may have reduced the statistical power of the multivariable analysis. Third, systemic inflammatory indices derived from routine blood counts can be influenced by multiple physiological and clinical factors, including metabolic control, medications, and systemic comorbidities. Fourth, although prior ocular treatment was considered, the one-month exclusion window may not fully eliminate its effects. Previous interventions, such as anti-VEGF or laser therapy, may have lasting impacts on retinal status, and residual confounding related to treatment and disease severity cannot be excluded. Therefore, the discriminatory performance of these markers should be interpreted carefully and requires validation in larger and independent cohorts.

## Conclusion

In conclusion, a multi-parametric approach incorporating NLR, SII, and BMI offers a reliable discriminatory tool for identifying DME in NPDR patients. These parameters are particularly valuable as they are highly accessible and cost-effective for routine exaination in diabetic retinal disease. While NPDR severity remains clinically relevant, its predictive value is optimized when integrated with these systemic metrics for a more holistic risk assessment. These findings provide a conceptual framework linking systemic metabolic status to retinal pathology and suggest that systemic inflammatory and metabolic conditions may contribute to the biological environment of DME. Larger prospective studies are warranted to further clarify the clinical utility of these biomarkers in diabetic retinal disease.

## Data Availability

The datasets used and/or analyzed during the current study are available from the corresponding author upon reasonable request.

## Abbreviations

AUC: Area under the curve
BMI: Body mass index
BCVA: Best–corrected visual acuity
CBC: Complete blood count
CMT: Central macular thickness
DME: Diabetic macular edema
DM: Diabetes mellitus
HbA1c: Glycated hemoglobin
IRF: Intraretinal fluid
MLR: Monocyte–to–lymphocyte ratio
NLR: Neutrophil–to–lymphocyte ratio
NPDR: Non–proliferative diabetic retinopathy
OCT: Optical coherence tomography
PLR: Platelet–to–lymphocyte ratio
PRP: Panretinal photocoagulation
ROC: Receiver operating characteristic
SII: Systemic immune–inflammation index
SPSS: Statistical package for the social sciences
SRF: Subretinal fluid
T2DM: Type 2 diabetes mellitus
VEGF: Vascular endothelial growth factor

## References

[1] Kuroiwa DAK, Malerbi FK, Regatieri CVS. New Insights in Resistant Diabetic Macular Edema. Ophthalmologica. 2021;244(6):485–94. 10.1159/000516614 PubMed PMID: 34023834. 34023834 10.1159/000516614

[2] Shah SU, Maturi RK. Therapeutic Options in Refractory Diabetic Macular Oedema. Drugs. 2017;77(5):481–92. 10.1007/S40265-017-0704-6 . PubMed PMID: 28197794. 28197794 10.1007/s40265-017-0704-6

[3] Browning DJ, Stewart MW, Lee C. Diabetic macular edema: Evidence-based management. Indian J Ophthalmol. 2018;66(12):1736–50. IJO.IJO_1240_18 PubMed PMID: 30451174. 30451174 10.4103/ijo.IJO_1240_18 PMC6256891

[4] Noma H, Mimura T, Yasuda K, Shimura M. Role of Inflammation in Diabetic Macular Edema. Ophthalmologica. 2014;232(3):127–35. 10.1159/000364955. PubMed PMID: 25342084. 25342084 10.1159/000364955

[5] Harley O, Amelia YS, Gustianty E, Soetedjo NNM, Kartasasmita AS. Retinal microglia: revealing new opportunities for identifying early biomarkers of diabetic retinopathy. Curr Eye Res. 2025. 10.1080/02713683.2025.2517300. 40555254 10.1080/02713683.2025.2517300

[6] Zhang J, Zhang J, Zhang C, Zhang J, Gu L, Luo D et al. Diabetic macular edema: current understanding, molecular mechanisms and therapeutic implications. Cells. 2022;11(21):3362. 10.3390/CELLS11213362 PubMed PMID: 36359761. 10.3390/cells11213362 PMC9655436 36359761

[7] Noma H, Yasuda K, Shimura M. Involvement of cytokines in the pathogenesis of diabetic macular edema. Int J Mol Sci. 2021;22(7):3427. 10.3390/IJMS22073427. PubMed PMID: 33810434. 10.3390/ijms22073427 PMC8036935 33810434

[8] Harley O, Amelia YS, Gustianty E, Soetedjo NNM, Kartasasmita AS. Exploring leukocyte differential count ratio profiles as inflammatory biomarkers in diabetic retinopathy: a systematic review and meta-analysis. BMC Ophthalmol. 2025;25(1). 10.1186/S12886-025-04075-Y . PubMed PMID: 40312699. 10.1186/s12886-025-04075-y PMC12044949 40312699

[9] Yang Z, Tan TE, Shao Y, Wong TY, Li X. Classification of diabetic retinopathy: Past, present and future. Front Endocrinol (Lausanne). 2022;13:1079217. 10.3389/FENDO.2022.1079217. /FULL PubMed PMID: 36589807. 36589807 10.3389/fendo.2022.1079217 PMC9800497

[10] Yang S, Berdine G. The Receiver Operating Characteristic (ROC) curve. 2017;5(19):34. 10.12746/SWRCCC.V5I19.391

[11] Harley O, Amelia YS, Gustianty E, Soetedjo NNM, Kartasasmita AS. Exploring leukocyte differential count ratio profiles as inflammatory biomarkers in diabetic retinopathy: a systematic review and meta-analysis. BMC Ophthalmol. 2025;25(1):265. 10.1186/S12886-025-04075-Y/TABLES/2 . PubMed PMID: 40312699. 40312699 10.1186/s12886-025-04075-y PMC12044949

[12] Dascalu AM, Serban D, Tanasescu D, Vancea G, Cristea BM, Stana D, et al. The value of white cell inflammatory biomarkers as potential predictors for diabetic retinopathy in Type 2 Diabetes Mellitus (T2DM). Biomedicines. 2023;11(8). 10.3390/BIOMEDICINES11082106. PubMed PMID: 37626602. 10.3390/biomedicines11082106 PMC10452280 37626602

[13] Bhutia CU, Kaur P, Singh K, Kaur S. Evaluating peripheral blood inflammatory and metabolic biomarkers as predictors in diabetic retinopathy and diabetic macular edema. Indian J Ophthalmol. 2023;71(6):2521–5. IJO.IJO_345_23 PubMed PMID: 37322673. 37322673 10.4103/IJO.IJO_345_23 PMC10417976

[14] Figueras-Roca M, Molins B, Sala-Puigdollers A, Matas J, Vinagre I, Ríos J, et al. Peripheral blood metabolic and inflammatory factors as biomarkers to ocular findings in diabetic macular edema. PLoS ONE. 2017;12(3):e0173865. 10.1371. /JOURNAL.PONE.0173865 PubMed PMID: 28328965. 28328965 10.1371/journal.pone.0173865 PMC5362077

[15] Nie Y, Zhou H, Wang J, Kan H. Association between systemic immune-inflammation index and diabetes: a population-based study from the NHANES. Front Endocrinol (Lausanne). 2023;14:1245199. 10.3389/FENDO.2023.1245199/BIBTEX. 38027115 10.3389/fendo.2023.1245199 PMC10644783

[16] Dogan L, Ozer Ö, Guclu E. The Effect of Systemic Inflammatory Biomarkers and Dyslipidemia on the Prognosis of Diabetic Retinopathy in Patients with Type 2 Diabetes Mellitus: Retrospective Research. Turkiye Klinikleri J Ophthalmol. 2024;33(4):219–28. 10.5336/OPHTHAL.2024-103341.

[17] Zhou T, Hu Z, Yang S, Sun L, Yu Z, Wang G. Role of adaptive and innate immunity in type 2 diabetes mellitus. J Diabetes Res. 2018;2018. 10.1155/2018/7457269 PubMed PMID: 30533447. 10.1155/2018/7457269 PMC6250017 30533447

[18] Xia C, Rao X, Zhong J. Role of T lymphocytes in type 2 diabetes and diabetes-associated inflammation. J Diabetes Res. 2017;2017. 10.1155/2017/6494795. PubMed PMID: 28251163. 10.1155/2017/6494795 PMC5307004 28251163

[19] Hameed I, Masoodi SR, Mir SA, Nabi M, Ghazanfar K, Ganai BA. Type 2 diabetes mellitus: From a metabolic disorder to an inflammatory condition. World J Diabetes. 2015;6(4):598. 10.4239/WJD.V6. I4.598 PubMed PMID: 25987957. 25987957 10.4239/wjd.v6.i4.598 PMC4434080

[20] Chen J, Tan W. Platelet activation and immune response in diabetic microangiopathy. Clin Chim Acta. 2020;507:242–7. 10.1016/J.CCA. .2020.04.042 PubMed PMID: 32376322. 32376322 10.1016/j.cca.2020.04.042

[21] Ogura S, Kurata K, Hattori Y, Takase H, Ishiguro-Oonuma T, Hwang Y, et al. Sustained inflammation after pericyte depletion induces irreversible blood-retina barrier breakdown. JCI Insight. 2017;2(3). 10.1172/.JCI.INSIGHT.90905. PubMed PMID: 28194443. 10.1172/jci.insight.90905 PMC5291729 28194443

[22] Huang H, Gandhi JK, Zhong X, Wei Y, Gong J, Duh EJ, et al. TNFα is required for late BRB breakdown in diabetic retinopathy, and its inhibition prevents leukostasis and protects vessels and neurons from apoptosis. Invest Ophthalmol Vis Sci. 2011;52(3):1336–44. 10.1167/IOVS.10-5768/-/DCSUPPLEMENTAL PubMed PMID: 21212173. 10.1167/iovs.10-5768 PMC3101693 21212173

[23] Wang S, Pan X, Jia B, Chen S. Exploring the Correlation Between the Systemic Immune Inflammation Index (SII), Systemic Inflammatory Response Index (SIRI), and Type 2 Diabetic Retinopathy. Diabetes Metabolic Syndrome Obes. 2023;16:3827–36. 10.2147/DMSO.S437580. 10.2147/DMSO.S437580 PMC10683512 38033457

[24] Ilhan C, Citirik M, Uzel MM, Kiziltoprak H, Tekin K. The usefulness of systemic inflammatory markers as diagnostic indicators of the pathogenesis of diabetic macular edema. Arq Bras Oftalmol. 2020;83(4):299–304. 10.5935/0004-2749.20200051 . PubMed PMID: 32756784. 32756784 10.5935/0004-2749.20200051 PMC11826592

[25] Yumnamcha T, Guerra M, Singh LP, Ibrahim AS. Metabolic dysregulation and neurovascular dysfunction in diabetic retinopathy. Antioxidants. 2020;9(12):1–22. 10.3390/ANTIOX9121244 10.3390/antiox9121244 PMC7762582 33302369

[26] Kowluru RA, Zhong Q, Kanwar M. Metabolic Memory and Diabetic retinopathy: Role of Inflammatory Mediators in Retinal Pericytes. Exp Eye Res. 2010;90(5):617. 10.1016/J.EXER. .2010.02.006 PubMed PMID: 20170650. 20170650 10.1016/j.exer.2010.02.006 PMC2857535

[27] Klop B, Elte JWF, Cabezas MC. Dyslipidemia in obesity: mechanisms and potential targets. Nutrients. 2013;5(4):1218–40. 10.3390/NU5041218 PubMed PMID: 23584084. 23584084 10.3390/nu5041218 PMC3705344

[28] Yue S, Zhang J, Wu J, Teng W, Liu L, Chen L. Use of the Monocyte-to-Lymphocyte Ratio to Predict Diabetic Retinopathy. Int J Environ Res Public Health. 2015;12(8):10009–19. 10.3390/IJERPH120810009 PubMed PMID: 26308022. 26308022 10.3390/ijerph120810009 PMC4555325

[29] Sari DA, Delfi, Virgayanti V, Sari MD. Evaluation of neutrophil-to-lymphocyte ratio, monocyte-to-lymphocyte ratio and platelet-to-lymphocyte ratio as predictor factors on diabetic retinopathy. Eur Mod Stud J [Internet]. 2021 Nov 22 [cited 2024 Dec 8];5(5):168–176. Available from: https://journal-ems.com/index.php/emsj/article/view/369

[30] Dascalu AM, Georgescu A, Costea AC, Tribus L, El Youssoufi A, Serban D, et al. Association between Neutrophil-to-Lymphocyte ratio (NLR) and Platelet-to-Lymphocyte Ratio (PLR) with diabetic retinopathy in type 2 diabetic patients. Cureus. 2023;15(11):e48628. 10.7759/cureus.48581. PubMed PMID: 38090430. 10.7759/cureus.48581 PMC10711340 38090430

[31] Harley O, Amelia YS, Gustianty E, Soetedjo NNM, Kartasasmita AS. Systemic–retinal inflammatory crosstalk in diabetic macular edema: correlation between hematologic indices and macular OCT-features. Int J Retina Vitreous. 2026;12(1):32. 10.1186/S40942-025-00794-Y/FIGURES/3. 41530827 10.1186/s40942-025-00794-y PMC12892436

[32] Zhou J, Song S, Zhang Y, Jin K, Ye J. OCT-Based Biomarkers are Associated with Systemic Inflammation in Patients with Treatment-Naïve Diabetic Macular Edema. Ophthalmol Ther. 2022;11(6):2153–67. 00576-X PubMed PMID: 36166152. 36166152 10.1007/s40123-022-00576-x PMC9587150

[33] Yanxia C, Xiongyi Y, Min F, Xiaoyun K. Optical Coherence Tomography-Based Grading of Diabetic Macular Edema Is Associated with Systemic Inflammatory Indices and Imaging Biomarkers. Ophthalmic Res. 2024;67(1):96–106. 10.1159/000535199 PubMed PMID: 38211574. 38211574 10.1159/000535199

